# CTLs From Patients With Atherosclerosis Show Elevated Adhesiveness and Distinct Integrin Expression Patterns on 2D Substrates

**DOI:** 10.3389/fmed.2022.891916

**Published:** 2022-07-13

**Authors:** Daria M. Potashnikova, Aleena A. Saidova, Anna V. Tvorogova, Alexandra S. Anisimova, Alexandra Yu Botsina, Elena Yu Vasilieva, Leonid B. Margolis

**Affiliations:** ^1^Laboratory of Atherothrombosis, Moscow State University of Medicine and Dentistry, Moscow, Russia; ^2^Moscow Department of Healthcare, City Clinical Hospital Named After I.V. Davydovsky, Moscow, Russia; ^3^Section on Intercellular Interactions, Eunice Kennedy Shriver National Institute of Child Health and Human Development, National Institutes of Health, Bethesda, MD, United States

**Keywords:** CTL, integrins, chemokines, atherosclerosis, T-lymphocyte adhesion, T-lymphocyte migration

## Abstract

Atherosclerosis is the major cause of cardiovascular disease that is characterized by plaque formation in the blood vessel wall. Atherosclerotic plaques represent sites of chronic inflammation with diverse cell content that is shifted toward the prevalence of cytotoxic T-lymphocytes (CTLs) upon plaque progression. The studies of CTL recruitment to atherosclerotic plaques require adequate *in vitro* models accounting for CTL interactions with chemokine-ligands and extracellular matrix fibers *via* surface chemokine receptors and integrins. Here we applied such a model by investigating CTL adhesion and migration on six types of coated surfaces. We assessed adhesion and motility metrics, the expression of chemokine receptors, and integrins in CTLs of patients with atherosclerosis and healthy donors. Using fibronectin, platelet-poor plasma from patients with atherosclerosis, and conditioned medium from atherosclerotic plaques we revealed the role of substrate in CTL adhesiveness: fibronectin alone and fibronectin combined with platelet-poor plasma and conditioned medium elevated the CTL adhesiveness – in patients the elevation was significantly higher than in healthy donors (*p* = 0.02, mixed 2-way ANOVA model). This was in line with our finding that the expression levels of integrin-coding mRNAs were elevated in the presence of fibronectin (*p* < 0.05) and ITGB1, ITGA1, and ITGA4 were specifically upregulated in patients compared to healthy donors (*p* < 0.01). Our experimental model did not affect the expression levels of mRNAs CCR4, CCR5, and CX3CR1 coding the chemokine receptors that drive T-lymphocyte migration to plaques. Thus, we demonstrated the substrate-dependence of integrin expression and discriminated CTLs from patients and healthy donors by adhesion parameters and integrin expression levels.

## Introduction

T-lymphocyte migration is a physiological process that occurs during normal maturation and also determines the immune response to a large number of stimuli in disease (1). As part of immune surveillance and inflammatory reaction of the organism different subtypes of T-lymphocytes are attracted to infected cells (2), tumors (3), and rheumatoid lesions (4) as well as to the vessel walls containing atherosclerotic plaques (5, 6). As the impact of T-cells on the pathogenesis of a variety of clinically important conditions becomes more evident, the focus of many studies is drawn to the specific mechanisms driving them to different organs and the phenotypic qualities of the attracted responder T-cell populations.

With atherosclerosis being a major cause of cardiovascular disease, and thus essentially accounting for the largest number of deaths and disabilities worldwide, the model of T-lymphocyte migration to atherosclerotic plaque calls for much attention. Atherosclerotic plaques that are often observed in the carotid artery wall and upon rapture trigger thrombosis, leading to myocardial infarction and stroke, present a site of chronic inflammation with heterogeneous immune cell content (7). Additional aberrant immune activation contributes to atherosclerosis progression (8–10) that is associated with activation and accumulation of T-lymphocytes in atherosclerotic plaques (11, 12). In the end T-lymphocytes and specifically CD8+ cytotoxic T-lymphocytes (CTLs) comprise the predominant population in advanced atherosclerotic plaques (12, 13) and although their functions are not completely clear, there is evidence of their role in plaque destabilization, confirmed on animal models (14).

The studies of cytotoxic T-lymphocyte migration to different sites employ various models including transwell and transgel (15, 16) as well as 2D assays (17), each of them have their advantages and limitations. The transwell assays focus on the chemotaxis analysis (a migration driven by a gradient of soluble chemokines, which occurs when an asymmetry in chemoattractant) to establish the role of chemokines, in CTL recruiting to tissues. The 2D models are used to explore haptokinesis (a migration along a surface, utilizing immobilized ligands such as chemokines or integrins, without any cue gradient to provide a directional bias) and subsequently the role of integrin adhesion in CTL migration. Usually, the cross-talk of the chemokine and integrin systems is not addressed in such models. Chemotaxis migration models in collagen (transgel) are considered more physiologic as they account for the presence of extracellular matrix and the integrin involvement in this process but do not allow to estimate of the differential effect of integrins on cell migration descriptors. Here we employ different 2D coatings to visualize the impact of extracellular matrix and the plaque-produced signaling molecules on CTLs of patients with atherosclerosis and healthy donors in the absence of specific antigen signaling. We assess the CTL adhesion properties and provide motility metrics along with the transcription level of the key migration-associated molecules–chemokine receptors and integrins. Together these findings will help to better understand the process of CTL migration into atherosclerotic plaques and gain new insight into the cellular mechanisms that drive it.

## Materials and Methods

### Patient Plasma and Conditioned Medium Samples

The study involving blood plasma and a conditioned culture medium of human participants was performed according to the Declaration of Helsinki and approved by the Interuniversity Committee of Ethics (Prot#11 16.12.2021). The patients/participants provided their written informed consent to participate in this study.

Peripheral blood samples and atherosclerotic plaques were obtained from symptomatic patients admitted to the Neurology Department of Clinical City Hospital named after I.V. Davydovsky with cardiovascular disease, most often thrombosis and stroke. All patients had atherosclerosis of the carotid artery. Some of the patients were forwarded to endarterectomy shortly after admission. Blood plasma and conditioned culture media from cultured endarterectomies plaques were collected from the patients and stored as follows:

Peripheral blood was obtained from patients prior to endarterectomy, collected into ficoll-containing BD Vacutainer CPT tubes (BD, USA), and processed <20 min after withdrawal. Initial centrifugation was performed at 2,000 g for 20 min. The upper fraction 10 mm above the ring of mononuclear cells was transferred to new tubes, pipetted, and used to obtain platelet-poor plasma. Platelet-poor plasma was obtained by the standard method: two rounds of centrifugation at 3,000 g for 15 min. After each centrifugation, the supernatant was transferred to new tubes without disturbing the pellet and carefully pipetted to avoid foaming. Frozen aliquots of platelet-poor plasma were stored at −80°C and thawed to coat the plastic wells for T-cell seeding.

Endarterectomized plaques were processed <2 h after the operation. Plaque specimens were washed in a cell culture medium to remove the contaminating blood-derived mononuclear cells, dissected, placed on a wetted collagen sponge raft (Pfizer, USA) at the medium–air interface, and cultured in AIM V serum-free medium (Thermo Fisher Scientific, USA) at 37°C/5% CO_2_ as described earlier (18). After 24 h of explant culture, the medium was changed. The plaque explants were then incubated for 72 h more, the culture medium was collected and centrifuged two times at 3,000 g for 15 min. After each centrifugation, the supernatant was transferred to new tubes without disturbing the pellet and carefully pipetted to avoid foaming. Frozen aliquots of conditioned culture medium were stored at −80°C and thawed to coat the plastic wells for T-cell seeding.

### T-Lymphocyte Sorting

**Figure 1:**
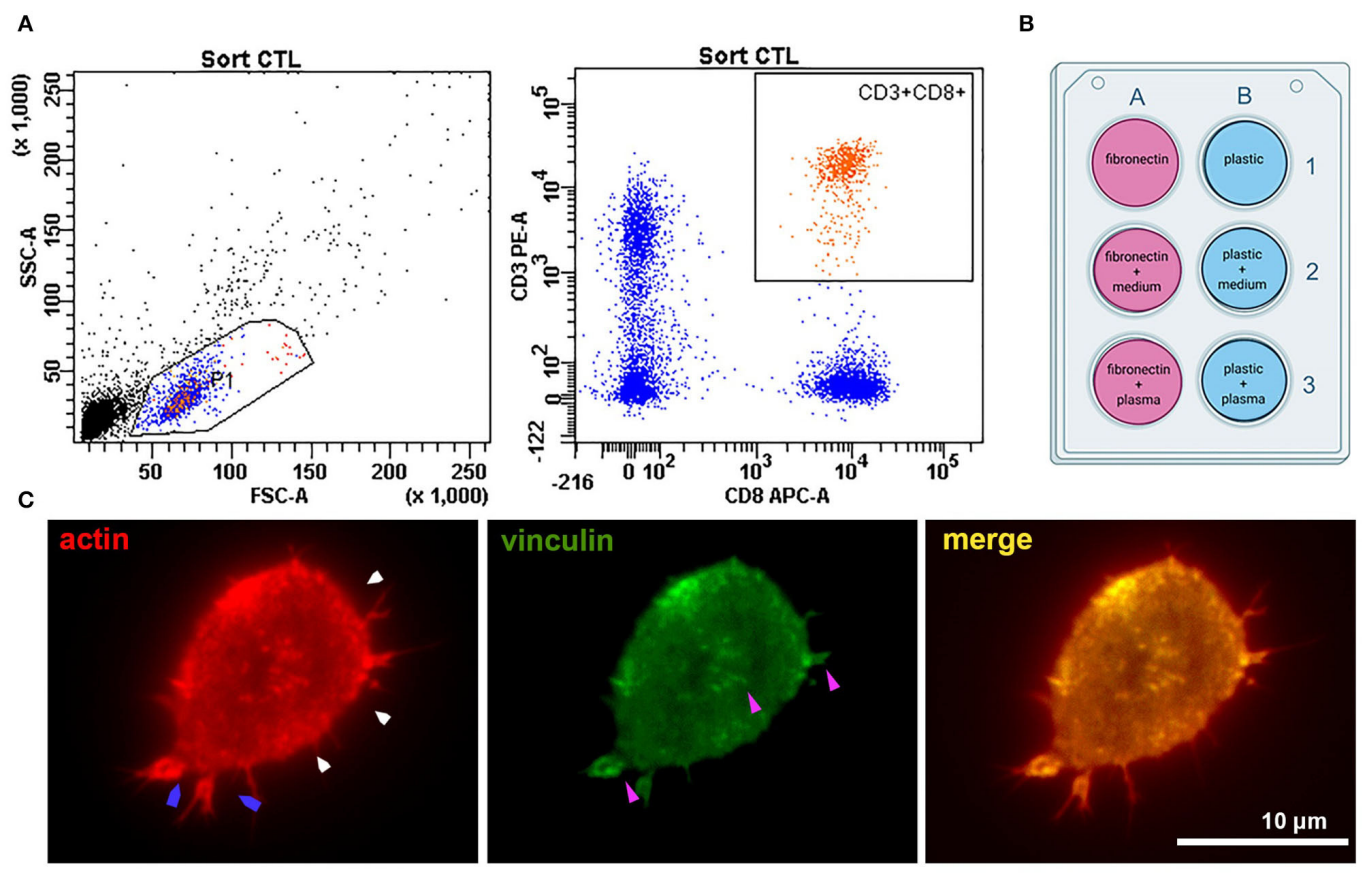
Experiment setup and cell adhesion metrics were calculated for all types of coating. **(A)** Sorting CTLs from peripheral blood; the gates are set on the CD3+CD8+ T-cell population. **(B)** The six types of surface coating tested for each experiment. Fibronectin coating was applied for 45 min at room temperature – 100 μl of 50 μg/ml solution per 1 cm^2^ surface. Fibronectin coating was air-dried before applying other ligands. Plasma and conditioned culture medium were stored after centrifugation at −80°C and thawed only once to coat the surfaces for T-cell seeding. Conditioned medium for coating in all experiments was derived from one batch (medium conditioned for 3 days by an atherosclerotic plaque explants from a male patient (age 61 years) with an ischemic stroke and atherosclerosis of carotid artery). Plasma was used from four different batches – one batch for each patient + donor pair (by number, as provided in Supplementary Table 1). All plasma was obtained from patients with confirmed atherosclerosis of carotid arteries and neurological symptoms (thrombosis and stroke) **(C)** Representative fluorescence image of a CD8+ T-lymphocyte seeded on a coverslip (patient 2, fibronectin coating) and stained against actin and vinculin after 24 h' incubation. The cell forms small lamellipodia (blue arrows), actin is organized into a thin cortical network (white arrows). Focal adhesions (purple arrows, visualized by anti-vinculin fluorescence staining) form spots mostly at the T-cell periphery.

Six samples of venous blood (three from healthy donors and three from patients with atherosclerosis) were collected into BD Vacutainer CPT tubes (BD, USA). Characteristics of healthy donors and patients are presented in Supplementary Table 1. Tubes were centrifuged at 2,000 g for 20 min. The ring of mononuclear cells was collected and washed twice in PBS (PanEco, Russia) at 1,000 g 15 min. Surface staining was performed in PBS with anti-CD3-APC (clone OKT3, BioLegend, USA) and anti-CD8a-PE (clone HIT8a, BioLegend, USA) antibodies for 20 min in the dark at RT. CD8+ T-lymphocytes were sorted using a FACSAria SORP cell sorter (BD Biosciences, USA) with a 70 um nozzle and corresponding pressure parameters. The sorting gate is presented in Figure 1A. Cells were collected into AIM V serum-free medium (Thermo Fisher Scientific, USA) and further cultured in it.

### Plastic and Glass Coating

Plastic wells and glass coverslips placed at the bottom of the wells were coated with plasma or conditioned medium in the presence or absence of fibronectin. Fibronectin (Sigma-Aldrich, USA) coating was performed on 24-well-plates according to Sigma protocol (45 min at room temperature – 100 μl of 50 μg/ml solution per 1 cm^2^ surface), the wells were air-dried. The additional coating was performed by placing 350 μl of thawed aliquots of patient plasma or conditioned AIM V medium from atherosclerotic plaques into the well. In 2 h, the medium was washed out with PBS (PanEco, Russia). The experimental setup with different types of coating is presented in Figure 1B.

### T-Lymphocyte Viability

For viability experiments sorted CD8+ T-cells were seeded at 50 × 10^3^ cells per well. Viability was assessed by flow cytometry using a FACSAria SORP cell sorter (BD Biosciences, USA) 24 h after seeding. Triple staining of cells was performed in 0.5 ml AIM V for 15 min in the dark using 100 nM TMRE (Thermo Fisher Scientific, USA), 1 μg/ml DAPI (Thermo Fisher Scientific, USA), and 240 μg/ml Annexin V-FITC (BioLegend, USA).

### Random Walk Assay

Sorted CD8+ T-cells were seeded at 25–30 × 10^3^ cells per well. After 24 h of incubation at 37°C/5% CO_2_, the medium was changed and time-lapse videos of cells were made for each well. In the random walk assay, we evaluated the total distance, cell velocity, and migration efficiency. Total distance was the displacement that a cell made during observation. Medium cell velocity was calculated by dividing the track distance by track time, and migration efficiency was calculated by dividing the total distance by track distance. At least 20 cells were analyzed per experimental condition for each donor/patient.

### Fluorescence Staining

Sorted CD8+ T-cells were seeded at 25–30 × 10^3^ cells on glass coverslips. After 24 h of incubation at 37°C/5% CO_2_, the medium was changed and cells were fixed on glass in 4% paraformaldehyde for 15 min at room temperature and washed in PBS 3 times. After permeabilization in 0.01% Triton- × 100 in PBS, cells were stained with antibodies.

For vinculin/actin staining the primary antibodies against vinculin (clone VLN01, Thermo Fisher Scientific, USA) was used at 1:100, 37°C for 60 min. Cells were washed in PBS 3 times and stained with secondary antibodies anti-mouse-Alexa488, at 1:100, 37°C for 60 min. Cells were washed in PBS 3 times and co-stained with phalloidin-Alexa555 (Thermo Fisher Scientific, USA) at 37°C for 60 min.

For integrin beta1/integrin alpha4 staining the directly conjugated anti-beta1-APC antibody was used at 1:100 (clone MAR4, BD, USA). For anti-alpha4 cells were stained with primary (clone D2E1 Cell Signaling) antibody at 1:100 and secondary antibody anti-rabbit-Cy2 (Sigma, USA) as described for vinculin.

After washing in PBS 3 times all specimens were mounted in Mowiol (Thermo Fisher Scientific, USA).

### RNA and cDNA

Sorted CD8+ T-cells were seeded at 50×10^3^ cells per well. After 24 h of incubation at 37°C/5%, CO_2_ (six types of experimental conditions) cells were taken for RNA isolation and RTqPCR. RNA was extracted from cell suspensions using RNeasy Mini Kit (Qiagen, USA) according to the manufacturer's instructions. RNA concentration was measured using a NanoPhotometer (Implen, Germany), and its purity was assessed according to the A260/A280 and A260/A230 ratios. cDNA was transcribed using the iScript Advanced cDNA synthesis kit (Bio-Rad Laboratories, USA) according to the manufacturer's instructions, and 50 ng of total RNA was taken into reaction.

### Primers and Real-Time PCR

Real-time qPCR was performed using a CFX96 (Bio-Rad Laboratories Inc., USA) cycler.

The relative amounts of integrin-coding mRNAs were detected using iTaq Universal SYBR Green Supermix (Biorad Laboratories Inc., USA). The reaction protocol included denaturation (95°C, 10 min) and 39 amplification cycles [95°C, 15 s; Ta°C (annealing temperature is provided in Supplementary Table 2), 30 s; and 72°C, 60 s]. All samples were processed in duplicate. One sample of cDNA put into each PCR run served as an inter-run calibrator for combining data into one experiment. Primer sequences are provided in Supplementary Table 2. Primers were purchased from “DNA-Synthesis” (Moscow, Russia). Primer specificity was confirmed by melting curve analysis. The Ct values were determined for real-time PCR curves by setting the threshold at 5 SD for each run. qPCR data were normalized according to Vandesompele et al. (19) using *UBC* and *HPRT1* as reference genes.

The relative amounts of chemokine receptor-coding mRNAs were detected using primers with TaqMan probes. Primers and probes were purchased from “DNA-Synthesis” (Moscow, Russia), all sequences are provided in Supplementary Table 3. The reaction protocol included denaturation (95°C, 5 min), followed by 50 amplification cycles (95°C, 10 s; Ta°C (annealing temperature is provided in Supplementary Table 3), 30 s; and 72°C, 40 s). All samples were processed in duplicate. The qPCR data were normalized according to Vandesompele et al. (19) using *UBC* and *YWHAZ* as reference genes.

### Imaging

Live imaging was performed on an inverted Zeiss AxioObserver fluorescence microscope operating under Zen 3.1 Blue Edition software with × 20/1.6 objective (phase contrast) at 36.5–37°C in AIM-V (Gibco, Thermo Fisher Scientific, Waltham, MA, USA) using Hamamatsu ORCA-Flash 4.0 V2 (Hamamatsu Photonics, Hamamatsu, Japan) with 30 s intervals between frames, each video took at least 2 h. Images were analyzed using ImageJ software (NIH). Cell tracks were measured using the integrated MTrackJ plugin. Based on cell track data in a random walk assay, we evaluated the final distance, cell velocity, and migration efficiency. On the raw time-lapse videos, we measured the number of attached cells during 100 frames as a percentage of attached cells to all cells per field of view and the average time of attachment for them.

To visualize the actin fibers and vinculin standard FITC/Cy-3 filter cubes were used. Fluorescence images of attached T-cells were processed using ImageJ and finalized using Adobe Photoshop (Adobe Systems, USA) software.

Confocal integrin beta1/integrin alpha4 visualization was performed using Zeiss LSM900 with Ex.640nm/650-700nm Airyscan for integrin beta1-APC and Ex.488nm/502–545 nm Airyscan for integrin alpha4-Cy2.

### Data Analysis

The data were analyzed and plotted using GraphPad Prism 7 software (GraphPad Software, USA). Further statistical analysis was performed in R (20) with basic package “stats” ver. 4.1.1 and additional packages “margins” ver. 0.3.26 (21) and “ggplot2” ver. 3.3.5 (22): to evaluate the differences in gene expression levels between samples in different experimental conditions generalized linear models (GLM) were constructed. Normalized mRNA expression level was used as the dependent variable and “status–donor/patient,” “surface–fibronectin/plastic,” “addition–plasma/no plasma” and “addition–medium/no medium”- as predictors. Optimal model structure selection was performed using a stepwise algorithm according to the Akaike criterion (AIC).

## Results

### Sorted CTLs Demonstrate Similar Cell Viability and Cell Morphology on Different Types of Coating

In each experiment CD8+ T-lymphocytes were sorted (Figure 1A) and seeded on 6 types of coatings (Figure 1B): 1 – non-coated plastic; 2 – plastic coated with conditioned medium from atherosclerotic plaque; 3 – plastic coated with plasma from patients with atherosclerosis; 4 – fibronectin-coated plastic (FN); 5 – fibronectin-coated plastic with conditioned medium from atherosclerotic plaque (FN+medium); 6 – fibronectin-coated plastic with plasma from patients with atherosclerosis (FN+plasma). The viability of sorted CTLs was not affected by the coating and non-viable cells did not exceed 10.2% (Supplementary Figure 1).

In all experimental conditions, cells retained a generally similar morphology with short protrusions; actin was organized into a thin cortical network, and focal adhesion sites were visible at the T-cell edge and as small spots in the T-cell body (visualized with antibodies to vinculin) (Figure 1C).

### CTLs From Patients With Atherosclerosis Demonstrate Higher Adhesiveness Compared to Healthy Donors

**Figure 2:**
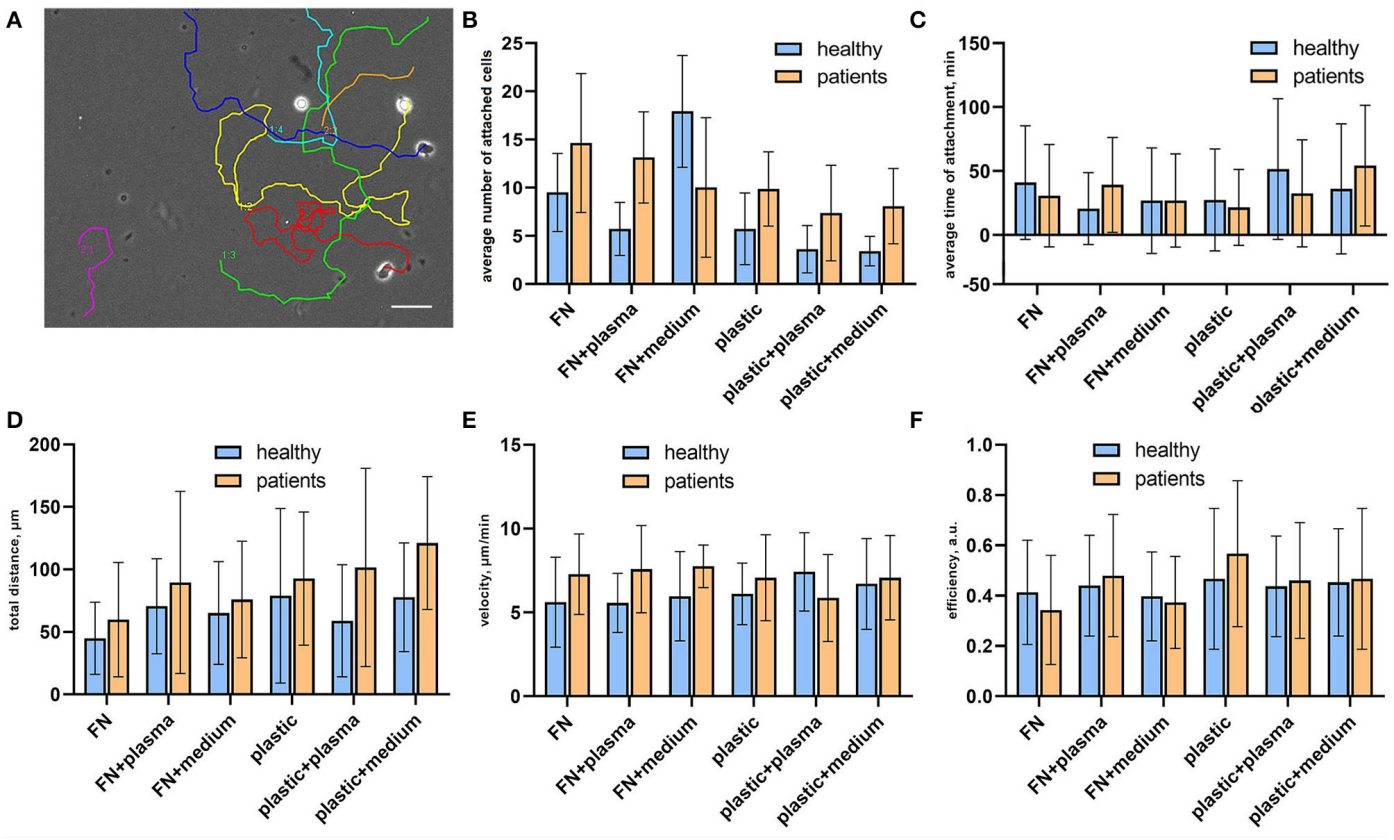
Random migration assay for sorted CTLs. **(A)** Representative migration tracks from a phase contrast time-lapse video summarized and plotted on one frame. Each video lasted for no <2 h with inter-frame interval of 30 s. **(B)** Cell adhesiveness in the tested conditions expressed as average number of attached cells per observation period. The data was obtained from phase contrast time-lapse videos. The number of attached cells was elevated in all samples from patients compared to healthy donors, the difference was statistically significant (*p* = 0.02, mixed 2-way ANOVA model) irrespective of substrate type or source of soluble ligands. **(C)** Cell adhesiveness in the tested conditions is expressed as the average time of attachment for attached cells. The time of the attachment was assessed as the time interval between the first frame with the cell attached to the substrate and the frame where it is detached. The data was obtained from phase contrast time-lapse videos. **(D)** Total migration distance of attached CTLs calculated as a displacement that a cell made during the time-lapse video, at least 20 cells were analyzed per experimental condition for each donor/patient. Each video lasted for no <2 h with an inter-frame interval of 30 s. **(E)** Medium cell velocity of attached CTLs calculated by dividing the track distance by track time. The data was obtained from phase contrast time-lapse videos, each video lasted for no <2 h with inter-frame interval of 30 s. At least 20 cells were analyzed per experimental condition for each donor/patient. **(F)** Migration efficiency of attached CTLs calculated by dividing the total distance by track distance. The data was obtained from phase contrast time-lapse videos, each video lasted for no <2 h with an inter-frame interval of 30 s. At least 20 cells were analyzed per experimental condition for each donor/patient.

To verify how surface coating affects the CTLs' adhesiveness and 2D migration parameters we analyzed attached cells and cell tracks in a random walk assay (Figure 2A). Cell adhesiveness was measured as the average number of cells attached to the substrate during the observation period and the average time they spent in the attached state. We found increased numbers of attached cells on fibronectin compared to non-coated plastic both for cells from patients and healthy donors (Figure 2B), except for cells attached on fibronectin with conditioned medium from atherosclerotic plaque. We found no differences in the percentage of attached cells in wells with added plasma and wells with added conditioned medium from atherosclerotic plaques, thus indicating that soluble ligands have no effect on T-cell adhesiveness in a 2D microenvironment. Most importantly, the percentage of attached cells was elevated in all samples from patients compared to healthy donors irrespective of substrate type or source of soluble ligands, the difference was statistically significant (*p* = 0.02, mixed 2-way ANOVA model). The average time of attachment was not affected by coating and was similar for CTLs from patients and healthy donors (Figure 2C).

### Cell Motility Parameters of CTLs Show a Trend for Upregulation in Patients With Atherosclerosis Compared to Healthy Donors

CTL tracks were further analyzed for cell motility parameters. The representative movies of motile CTLs with tracks are provided in Supplementary materials (Supplementary Videos 1–3). To describe the parameters of randomly migrating cells we measured the total displacement that a cell made during the observation time (“total distance”), as well as cell velocity and migration efficiency. Migration efficiency was calculated by dividing the total distance by track distance. A representative migration track with plotted track distance and the total distance is shown in Supplementary Figure 2. Mean values and distributions of migration descriptors were almost similar on different substrates. We observed a trend for the elevation of mean values of total distance and velocity for CTLs from patients in all conditions, thus indicating that CTLs from patients moved slightly faster than CTLs from healthy donors and thus could migrate to longer distances (although due to large ranges these differences were not statistically significant) (Figures 2D,E). The migration efficiency coefficients were almost similar for all conditions, indicating that neither CTLs from patients, nor CTLs from healthy donors could move persistently in the absence of a ligand gradient (Figure 2F). Cell velocity weakly correlated with the migration efficiency (Spearman R = 0.28) and total distance (R = 0.32), whereas the total distance moderately correlated with the migration efficiency (R = 0.49). This shows that fast-moving cells don't necessarily move farther, but rather cells that can maintain directional movement.

### mRNA Expression of ITGB1, ITGA1, and ITGA4 Is Upregulated in CTLs From Patients With Atherosclerosis Compared to Healthy Donors

**Figure 3:**
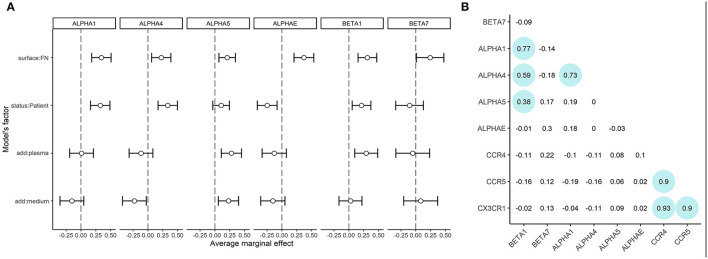
mRNA expression of integrin subunits and in CTLs **(A)** Integrin mRNA expression. Average marginal effects (AME) for linear generalized model, where zero states are CTLs from healthy donors, seeded on plastic without soluble ligands from plasma or conditioned medium from atherosclerotic plaques. The changed (model) factors are: (1) the status of the individual: donor/patient with atherosclerosis, (2) use of fibronectin in coating; (3) use of conditioned medium from atherosclerotic plaque in coating; (4) use of plasma from patients with atherosclerosis in coating. The dependent variable is the expression of integrins beta1, beta7, alpha1, alpha4, alpha5 or alphaE mRNAs.*ITGB1, ITGB7, ITGA1, ITGA4, ITGA5, and ITGAE* are upregulated (dependent, *p* < 0.05) in the presence of fibronectin. *ITGB1, ITGA1, ITGA4* are upregulated (dependent, *p* < 0.05) in patients compared to healthy donors. *ITGAE* is downregulated (dependent, *p* < 0.05) in patients compared to healthy donors. *ITGB1 and ITGA5* are upregulated (dependent, *p* < 0.05) in the presence of plasma. *ITGA5* is upregulated (dependent, *p* < 0.05) and *ITGA4* are downregulated (dependent, *p* < 0.05) in the presence of a conditioned medium from atherosclerotic plaque. **(B)** Spearman coefficients of correlation for mRNA expression data. The significant correlations (*p* < 0.05) are outlined in circles. A strong positive correlation is observed between *CCR5, CCR4, and CX3CR1* mRNAs expression. The mRNAs expression of *ITGB1, ITGA1, and ITGA4* also show positive correlation, additionally, *ITGB1* shows a weaker correlation with *ITGA5*.

As lymphocytes from healthy donors and patients demonstrated different adhesion capacities both on plastic and fibronectin, we evaluated the adhesion profile of CD8+ T-cells by measuring the expression of mRNAs coding α1, α4, α5, αE, β7, and β1 integrins (as the most common integrin subunits of CD8+ T-lymphocytes). The relative normalized expression levels are provided in Supplementary Figure 3. We assessed the role of fibronectin coating on integrin gene expression by comparing fibronectin-containing and non-containing experimental setups in a pairwise manner using a Wilcoxon test. Comparisons were made in groups of patients and healthy donors separately. The differences were statistically significant (*p* < 0.05) for *ITGB1, ITGA1, ITGAE* for healthy donors, and *ITGB1, ITGA1, ITGA4, and ITGAE* for patients. We have thus proved that substrate enhances the expression of specific integrin patterns in patient and donor CTLs. Most notably, we observed a substantial upregulation of integrin subunits in CTLs from patients with atherosclerosis compared to those from healthy donors. To estimate the precise contribution of each experimental condition (independent variables: patient/donor status, fibronectin in the coating, addition of plasma, the addition of conditioned medium) on integrin subunit mRNA expression (dependent variables) we built the generalized linear model of our assay. Using this model (Figure 3A, model coefficients and average marginal effect (AME) values are presented in Supplementary Table 4), we confirmed that fibronectin positively regulates the expression of all integrin subunits (AME from 0.20 to 0.37, *p* < 0.05). Moreover, mRNA expression of *ITGA1, ITGA4, and ITGB1* are upregulated (AME from 0.21 to 0.33, *p* < 0.01), and *ITGAE* is downregulated (AME −0.24, *p* < 0.01) in patients compared to healthy donors. We also showed the addition of plasma significantly upregulated *ITGA5, and ITGB1* (AME from 0.28 to 0.29, *p* < 0.005), while the addition of conditioned medium from plaque upregulated *ITGA5* (AME 0.23, *p* < 0.01) and downregulated *ITGA4* (AME −0.23, *p* < 0.03). Spearman correlation analysis (Figure 3B) revealed a group of co-expressed integrin mRNAs: *ITGA1, ITGA4, ITGB1*, and *ITGA5* in CTLs. We evaluated the mRNA levels of chemokine receptors typically present on blood CD8+ CTLs (Supplementary Figure 4). The mRNAs coding CCR4, CCR5, and CX3CR1 were all expressed at high levels, did not differ significantly between patients and healthy donors, and did not depend on the coating type as assessed in the generalized linear model (data not shown). However, we observed a strong positive correlation between the levels of these mRNAs in the whole sample set (Spearman coefficient >0.9, Figure 3B).

CTLs were immunostained with anti-β1 and anti-α4 in all experimental conditions followed by confocal imaging. No correlation of morphologic features or integrin distribution with experimental conditions was observed. However, in all cases, the β1 and α4 integrins were localized on the plasma membrane as shown by maximal intensity projection images and co-localized at the sites of focal adhesions (Supplementary Figures 5A–L).

## Discussion

T-lymphocytes are among the most motile cells in the human organism with a maximum *in vivo* velocity of 25 um/min (23) measured for migration within the lymph node and shown to be integrin-independent (24, 25). In this study, we sought to work out the model of integrin-dependent CTL migration that would allow assessment of their adhesion/migration metrics and to evaluate the expression levels of integrins and chemokine receptors in it. The model of migration on rigid substrate fibers accounts for the integrin switch that takes place in transendothelial (26) and intra-tissue migration of some T-cell populations during inflammation (27) *in vivo*. It allows the assessment of the resulting alterations in cell motility descriptors and is thus relevant *in vitro* model of plaque infiltration.

We used fibronectin coating as a source of extracellular matrix fibers and patient plasma/conditioned medium as additional sources of ligands associated with atherosclerosis. There is a lot of evidence that the blood plasma of patients with atherosclerosis is enriched in activation molecules, including chemokines which can drive CTL migration, e.g., CCL5, CCL4, CCL3, CX3CL1 (28, 29). A similar pattern of chemokines has been detected in atherosclerotic plaque lysates and in conditioned media from plaque explants (18, 29), although due to some differences in chemokine content we decided to use plasma and conditioned medium as two independent sources of stimulatory molecules. To analyze the activation status of CTLs in our assay, we measured the mRNA expression levels of chemokine receptors that were justified in previous studies: We and other authors showed that CCR4, CCR5, and CX3CR1 chemokine receptors are differentially expressed on T-lymphocytes in atherosclerotic plaques compared to peripheral blood T-lymphocytes and that mRNAs of *CCL2, CCL3*, and *CX3CL1* that code chemokine ligands to these receptors are expressed in plaques (30, 31). We observed a strong positive correlation of these receptors' expression both in CTLs from patients and healthy donors and that did not depend on the experimental conditions in this study. T-cell adhesion and migration were shown to be independently regulated in other models involving cell cultures and mice (32). This may also be the case for human *ex vivo* CTLs as is seen from the lack of correlation between the chemokine receptors' and integrins' expression.

To analyze the expression patterns of adhesion molecules in our assay we measured the mRNA expression levels of *ITGA1, ITGA4, ITGA5, ITGAE, ITGB1, and ITGB7* that encode the α1, α4, α5, αE, β1, and β7 integrins. As expected, in our model we observed the substantial upregulation of α4, α5, and β1 in CTLs plated on fibronectin, as it is an activation ligand for these molecules, but other integrins were also upregulated on fibronectin coating compared to non-coated plastic, possibly due to non-specific activation. It is worth noting that integrins are capable of promoting taxis as well as adhesion: as co-stimulatory molecules, some integrins may also play a role in T-lymphocyte activation and migration by binding glycoproteins on endothelial cells: thus CTLs may be co-stimulated by β1 integrin-dependent interaction with fibronectin (33, 34). To further understand the cross-talk between the integrins and chemokine receptors a signaling gradient must be added to the model.

Using the generalized linear model we showed that the expression levels of *ITGA1, ITGA4, and ITGB1* were elevated in CTLs from patients compared to healthy donors, and also that *ITGA5, ITGB1* could be elevated by the addition of plasma and/or conditioned medium from atherosclerotic plaques thus indicating the integrin patterns for CTLs of patients with atherosclerosis. This is important as the current works on gene expression report differential signaling, and pro-inflammatory and immune regulation-associated gene patterns for both bloods- and plaque-derived cells [30, 35]. No specific plaque-infiltrating phenotype [by analogy with skin-infiltrating and gut-infiltrating phenotypes (35)] that would include both integrins and chemokine receptors has been reported for these CTLs so far. The upregulated α4β1 and α1β1 integrins are known to provide the firm adhesion to endothelial cells before T-cell transmigration (36, 37), so their upregulation could be expected for CTLs of patients with atherosclerosis. To assess the integrin expression/localization at the protein level, we visualized β1 and α4 integrins in patient and donor CTLs. In all experimental conditions, the subunits were localized on the plasma membrane and co-localized at the sites of focal adhesions, although no significant differences in FA morphology were found using confocal imaging.

However, in our assay, the CTLs from patients with atherosclerosis showed significantly elevated adhesiveness. Also, CTLs from patients moved slightly faster and further than CTLs from healthy donors, but to prove the statistically significant differences high-throughput microscopy should be applied. In line with other experiments, we showed that CTLs could not maintain directional movement without the gradient of soluble ligands (38).

In conclusion, our study demonstrates the common effect of substrate on all integrins and justifies the use of extracellular fibers in CTL adhesion/migration experiments. On the other hand, it highlights a number of specific integrins that have elevated expression in CTLs of patients with atherosclerosis compared to healthy donors (*ITGA1, ITGA4, ITGB1*) and integrins that are upregulated in contact with the plasma of patients with atherosclerosis and/or conditioned media from atherosclerotic plaques (*ITGA5, ITGB1*). Such elevation may contribute to their enhanced adhesiveness and be of clinical importance in view of their plaque infiltration capacity *in vivo*.

## Data Availability Statement

The original contributions presented in the study are included in the article/Supplementary Material, further inquiries can be directed to the corresponding author.

## Ethics Statement

The studies involving human participants were reviewed and approved by Interuniversity Committee of Ethics, Moscow (Prot #11 16.12.2021). The patients/participants provided their written informed consent to participate in this study.

## Author Contributions

DP, AS, and AT conceived and performed the experiments, analyzed the data, and wrote the manuscript. AA and AB provided clinical material and summarized the clinical data. LM and EV conceived the experiments, analyzed the data, and edited the manuscript. All authors contributed to the article and approved the submitted version.

## Funding

The work was supported by the RSF grant #20-75-10085.

## Conflict of Interest

The authors declare that the research was conducted in the absence of any commercial or financial relationships that could be construed as a potential conflict of interest.

## Publisher's Note

All claims expressed in this article are solely those of the authors and do not necessarily represent those of their affiliated organizations, or those of the publisher, the editors and the reviewers. Any product that may be evaluated in this article, or claim that may be made by its manufacturer, is not guaranteed or endorsed by the publisher.
