# Supplementary material for: CTLs From Patients With Atherosclerosis Show Elevated Adhesiveness and Distinct Integrin Expression Patterns on 2D Substrates
Source: Front Med (Lausanne). 2022 Jul 13;9:891916. doi: 10.3389/fmed.2022.891916 (PMC9328274; doi:10.3389/fmed.2022.891916)
Supplement: Supplementary file 1 [file Data_Sheet_1.PDF]

Supplementary Material

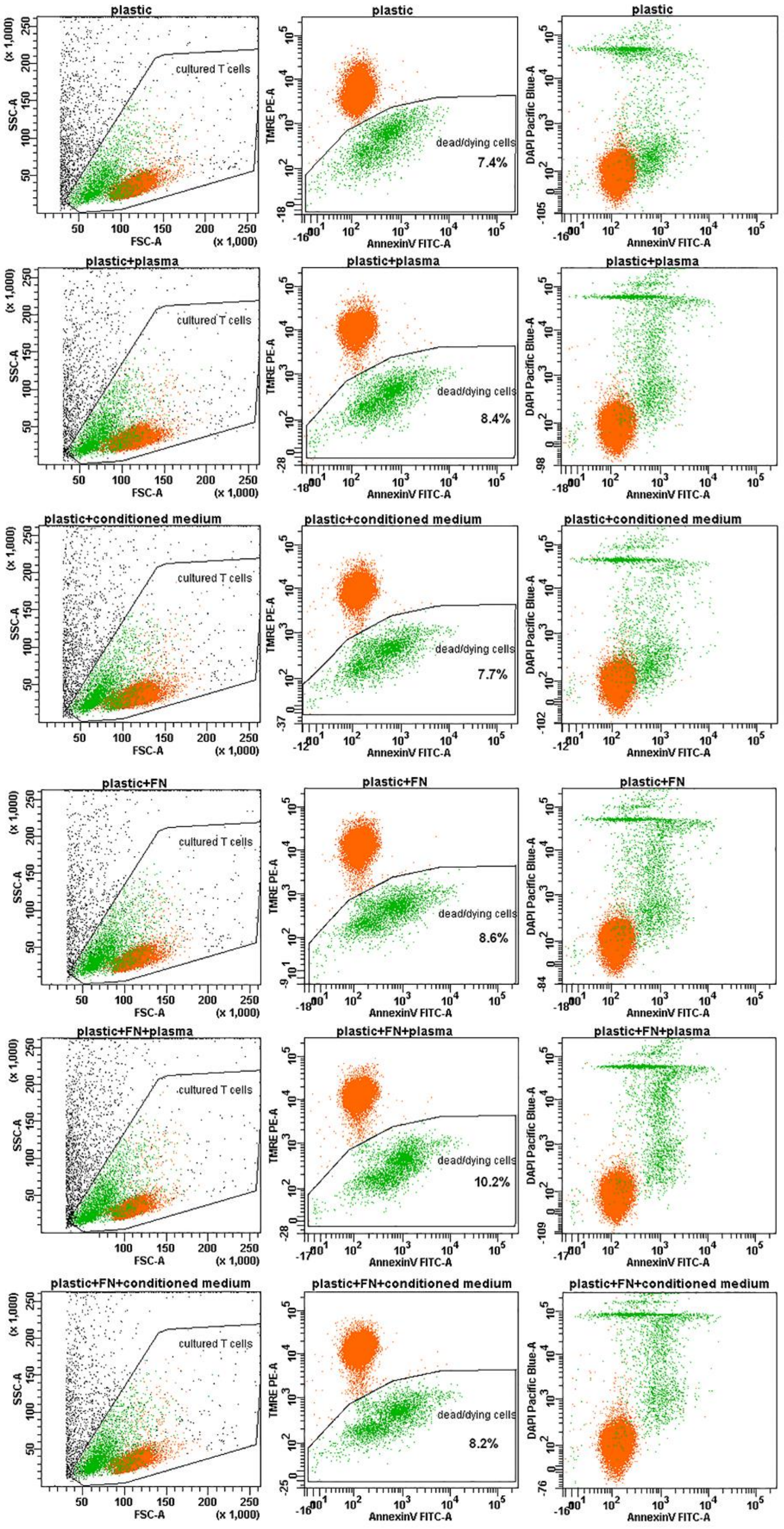

Supplementary Figure S1. The representative experiment on sorted CTLs' viability after 24 hours of incubation. Experimental conditions for each sample were as follows: 1) cells plated on plastic; 2) cells plated on plastic with added plasma from patients with atherosclerosis (plastic + plasma) 3) cells plated on plastic with added conditioned medium from atherosclerotic plaques (FN + conditioned medium); 4) cells plated on fibronectin (FN); 5) cells plated on fibronectin with added plasma from patients with atherosclerosis (FN + plasma); 6) cells plated on fibronectin with added conditioned medium from atherosclerotic plaques (plasma+ conditioned medium). Cells were stained with DAPI (a DNA dye, non-permeable for live cells), TMRE (as a mitochondrial potential-dependent dye) and Annexin V-Alexa488 (as a marker of phosphatidyl serine externalization) and analyzed using flow cytometry. Plotted for each condition are the light scatter parameters, TMRE vs Annexin V and DAPI vs Annexin V. The viable cells are TMREhigh/DAPI-/AnnexinV-; they are plotted in orange. The non-viable cells are TMRElow/DAPI+-/AnnexinV+-; they are plotted in green with percentages of the population.

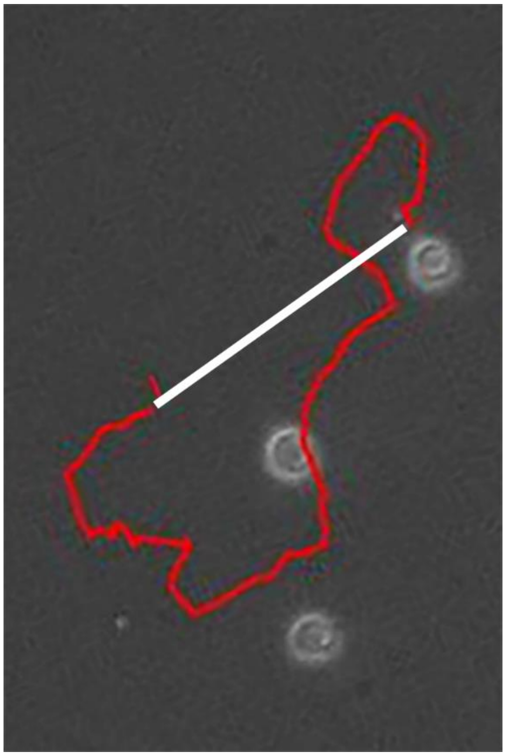

Supplementary Figure S2. A representative track for a migrating T-cell in a random walk 2D model (donor 3, fibronectin coating). The track distance (outlined in red) is assessed by plotting the position of the cell center in a sequence of frames with the inter-frame interval of 30 seconds. The total distance (outlined in white) is defined as the displacement of the cell during the observation time. The migration efficiency is calculated by dividing the total distance by the track distance.

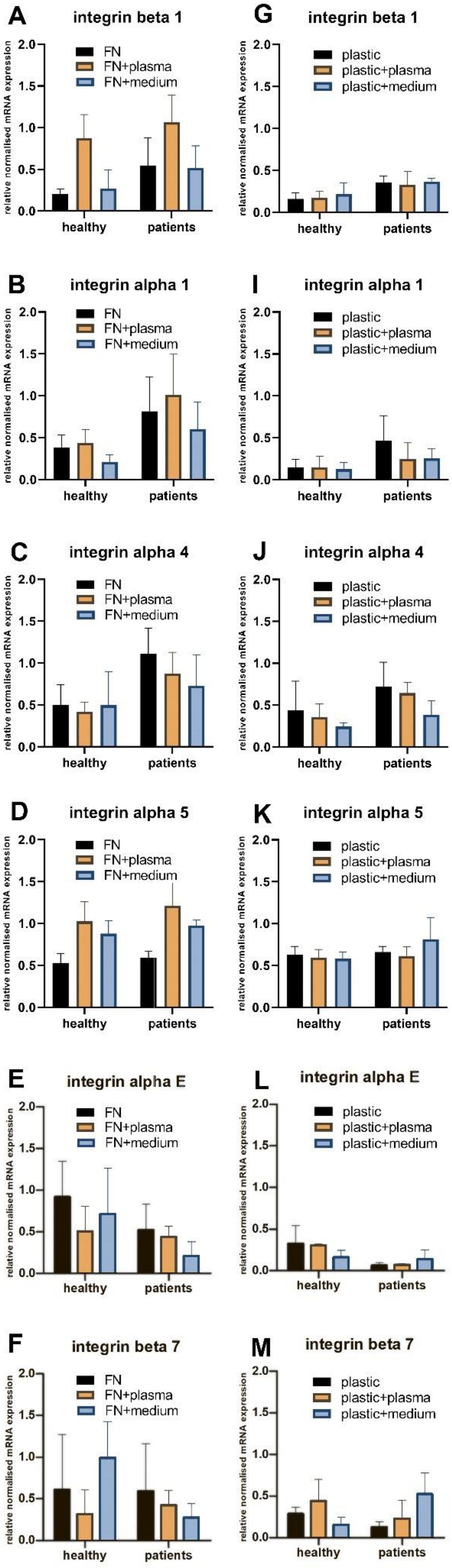
A
integrin beta 1
G
integrin beta 1
B
integrin alpha 1
I
integrin alpha 1
C
integrin alpha 4
J
integrin alpha 4
D
integrin alpha 5
K
integrin alpha 5
E
integrin alpha E
L
integrin alpha E
F
integrin beta 7
M
integrin beta 7
FN
FN+plasma
FN+medium
plastic
plastic+plasma
plastic+medium
relative normalised mRNA expression
0.0
0.5
1.0
1.5
2.0
healthy
patients

Supplementary Figure S3. mRNA expression levels of integrin subunits in CTLs from patients with cardiovascular disease (n=3) compared to CTLs from healthy donors (n=3). (**A-F)** experimental conditions for each sample were as follows: 1) cells plated on fibronectin (FN); 2) cells plated on fibronectin with added plasma from patients with atherosclerosis (FN + plasma); 3) cells plated on fibronectin with added conditioned medium from atherosclerotic plaque (FN + medium); (**G-M**) experimental conditions for each sample were as follows: 4) cells plated on plastic; 5) cells plated on plastic with added plasma from patients with atherosclerosis (plastic + plasma); 6) cells plated on plastic with added conditioned medium from atherosclerotic plaque (plasma + medium). RTqPCR reactions were set in duplicate, the data were normalized to *UBC* and *HPRT1* reference genes, the data are presented as average (boxes) and SD (whiskers).

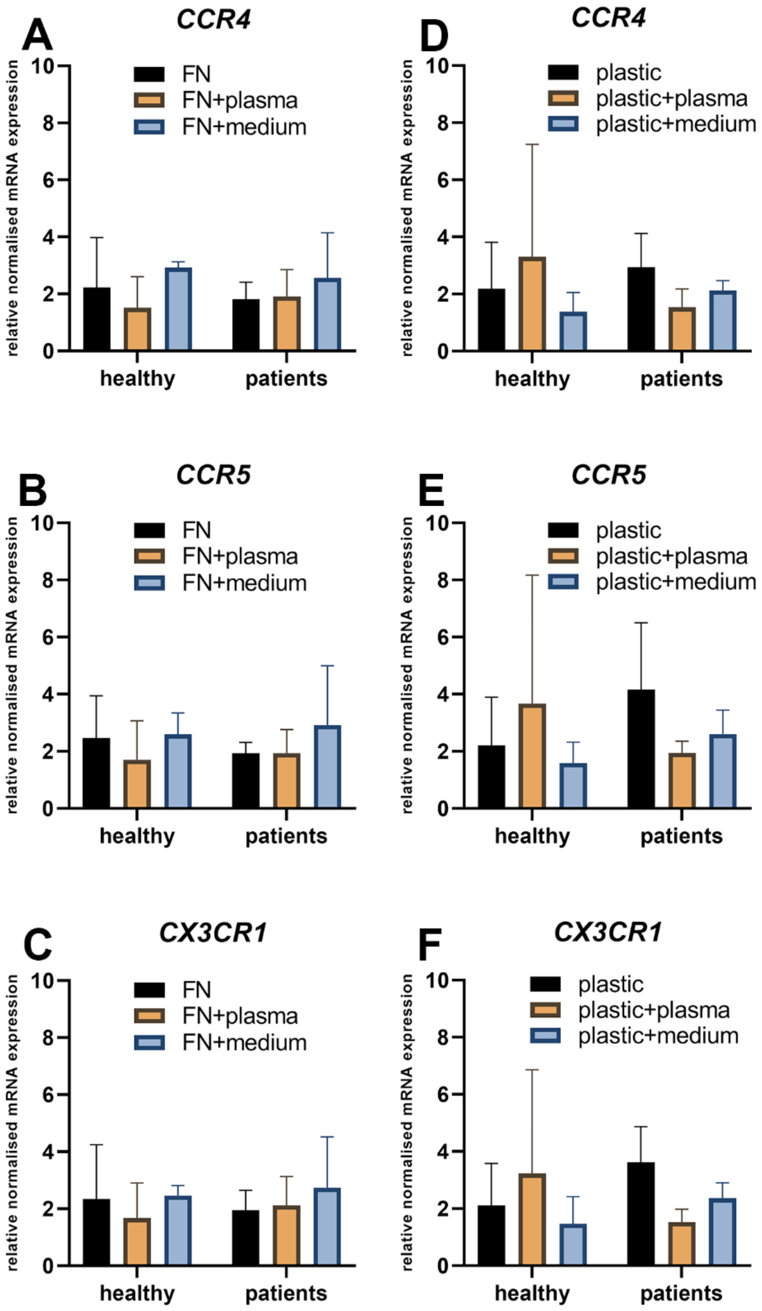

Supplementary Figure S4. mRNA expression levels of chemokine receptors in CTLs from patients with cardiovascular disease (n=3) compared to CTLs from healthy donors (n=3). **(A-C)** experimental conditions for each sample were as follows: 1) cells plated on fibronectin (FN); 2) cells plated on fibronectin with added plasma from patients with atherosclerosis (FN + plasma); 3) cells plated on fibronectin with added conditioned medium from atherosclerotic plaque (FN + medium); **(D-F)** experimental conditions for each sample were as follows: 4) cells plated on plastic; 5) cells plated on plastic with added plasma from patients with atherosclerosis (plastic + plasma); 6) cells plated on plastic with added conditioned medium from atherosclerotic plaque (plasma + medium). RTqPCR reactions were set in duplicate, the data were normalized to *UBC* and *YWHAZ* reference genes, the data are presented as average (boxes) and SD (whiskers).

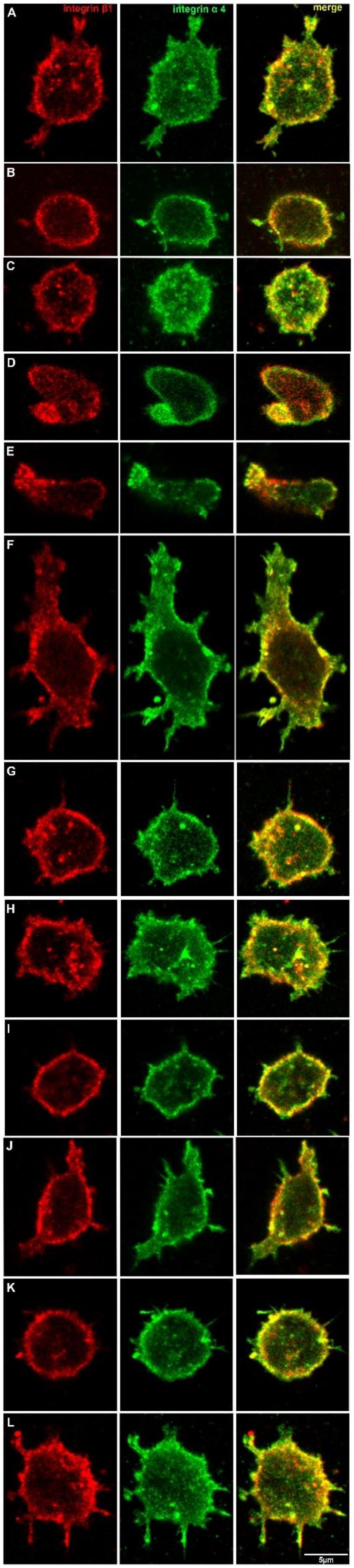

A
Integrin β1
Integrin α 4
merge
B
C
D
E
F
G
H
I
J
K
L
5μm

Supplementary Figure S5. Integrin α4 and β1 localization patterns for CTLs in different experimental conditions. Various types of cell morphology were observed: from cells forming multiple short protrusions to attached cells with a prominent leading edge. No correlations of morphologic features or integrin distribution with experimental conditions were found. However, in all cases the α4 and β1 integrins were localized on the plasma membrane, as is shown by maximal intensity projection images, and co-localized at the sites of focal adhesions. **A** – healthy donor, non-coated plastic; **B** – healthy donor, plastic with conditioned medium from atherosclerotic plaque; **C** - healthy donor, plastic with plasma from patients with atherosclerosis; **D** – healthy donor, fibronectin-coated plastic; **E** – healthy donor, fibronectin-coated plastic with conditioned medium from atherosclerotic plaque; **F** – healthy donor, fibronectin-coated plastic with plasma from patients with atherosclerosis; **G** – patient, non-coated plastic; **H** – patient, plastic with conditioned medium from atherosclerotic plaque; **I -** patient, plastic with plasma from patients with atherosclerosis; **J** – patient, fibronectin-coated plastic; **K** – patient, fibronectin-coated plastic with conditioned medium from atherosclerotic plaque; **L** – patient, fibronectin-coated plastic with plasma from patients with atherosclerosis.

Supplementary Table S1. Patient and donor information.

	Patient 1	Patient 2	Patient 3	Patient 4	Donor 1	Donor 2	Donor 3	Donor 4
Sex (M/F)	M	M	M	F	M	F	F	F
Age, years	64	70	55	66	31	47	37	39
CVD	I63.0 Ischemic stroke, atherotrombotic	I63.2 Ischemic stroke, atherotrombotic	G45.9 TIA	I65.2 Post-ischemic stroke, atherotrombotic	-	-	-	-
Arterial hypertension	Stage 3	NA	Stage 3	Stage 3	-	-	-	-
Atherotrosclerosis of carotid artery	+	+	+	+	-	-	-	-
Periperal atherosclerosis	+	-	NA	+	-	-	-	-
Endarterectomy	+	+	-	+	-	-	-	-

The median age for Patient group is 65 years, the median age for Donor group is 38 years. Sorted CTLs from Patients and Donors of the same number were processed in parallel. CTLs from Patients 1-3 and Donors 1-3 were used for live cell imaging, fluorescence actin/vinculin imaging and mRNA expression analysis. CTLs from Patient 4 and Donor 4 were used for integrin staining and confocal imaging.

Supplementary Table S2. Primer sequences for RTqPCR evaluation of integrin-coding mRNAs

Gene symbol	Forward and reverse primer sequence	Amplicon size	Sequence accession number	Ta,ºC
HPRT1	TGACACTGGCAAAACAATGCA<br>GGTCCTTTTCACCAGCAAGCT	94	NM_000194.3	61
UBC	ATTTGGGTCGCAGTTCTTG<br>TGCCTTGACATTCTCGATGGT	133	NM_021009.7	61
ITGB1	GGATTCTCCAGAAGGTGGTTTCG<br>TGCCACCAAGTTTCCCATCTCC	143	NM_002211.4	61
ITGA1	CCGAAGAGGTACTTGTTGCAGC<br>GGCTTCCGTGAATGCCTCCTTT	107	NM_181501.2	61
ITGA4	GCATACAGGTGTCCAGCAGAGA<br>AGGACCAAGGTGGTAAGCAGCT	116	NM_000885.6	60
ITGA5	GCCGATTCACATCGCTCTCAAC<br>GTCTTCTCCACAGTCCAGCAAG	139	NM_002205.5	60
ITGAE	CGTCCCAACCAAATTACGAG<br>CTTCCACATGCTGAACCGAA	128	NM_002208.5	60
ITGB7	GAAGGACTGCTCTGCACGC<br>ATCCCAAGCCGTAGTGGTAG	142	NM_000889.3	60

All oligonucleotides were synthesized by “DNA-Synthesis” (Russia). Sequences are presented in 5’-3’ direction.

Supplementary Table S3. Primer and probe sequences for RTqPCR evaluation of mRNAs coding chemokine receptors

Gene symbol	Forward primer, reverse primer<br>and probe sequences	Sequence accession number	Ta,ºC
UBC	TTGGGTCGCAGTTCTTGTTTG<br>TGCCTTGACATTCTCGATGGT<br>VIC-TCGCTGTGATCGTCACTTGACAATG-BHQ2	NM_021009.7	58
YWHAZ	GCAGGCTGAGCGATATGATG<br>ACCTACGGGCTCCTACAACA<br>ROX-GCATGAAGTCTGTAACTGAGCAAGGA-BHQ2	NM_001135700	58
CX3CR1	AGTGTGACTGAGACGGTTGC<br>AACACTTCCATGCCTGCTCC<br>VIC-CATTTGCTGGGGAGAAGTTCAGAAG-BHQ2	NM_001171171.2	58
CCR5	ACAGGGCTGTGAGGCTTATC<br>TCACCTGCATAGCTTGGTCC<br>VIC-CCTACAACATTGTCCTTCTCCTGAAC-BHQ2	NM_000579	58
CCR4	GGGTCATCACCAGTTTGGCT<br>GAGCTGAGAACCTTCCACG<br>ROX-TGGCTGTGTTCGCCTCCCTTC-BHQ2	NM_005508.5	58

All oligonucleotides were synthesized by "DNA-Synthesis" (Russia). Sequences are presented in 5'-3' direction. VIC – 2′-chloro-7′phenyl-1,4-dichloro-6-carboxy-fluorescein; ROX – carboxyrhodamine; BHQ2 – Black Hole Quencher 2.

Supplementary Table S4. Coefficients, average marginal effects and p-values for generalized linear model, where zero state are CTLs from healthy donors, plated on plastic without soluble ligands in medium. The changed (model) factors are: 1) the status of the individual: donor/patient; 2) use of fibronectin in coating; 3) use of conditioned medium from atherosclerotic plaque in coating; 4) use of plasma from patients with atherosclerosis in coating. The dependent variables are the expression of integrins beta1, beta7, alpha1, alpha4, alpha5 or alphaE. The graphic results are presented in Figure 3A.

**integrin alpha1**							marginal effects	
	Estimate	Std. Error	t value	Pr(>|t|)		factor	AME	SE
(Intercept)	0.117332	0.09179	1.278	2.11E-01				
statusp	0.325129	0.082099	3.96	0.000408	***	statusp	0.3251	0.0821
surface1fn	0.344816	0.082099	4.2	0.000209	***	surface1fn	0.3448	0.0821
addmedium	-0.153514	0.100551	-1.527	0.136968		addmedium	-0.1535	0.1006
addplasma	0.009465	0.100551	0.094	0.925612		addplasma	0.0095	0.1006
**integrin alpha4**							marginal effects	
	Estimate	Std. Error	t value	Pr(>|t|)		factor	AME	SE
(Intercept)	0.4122	0.0914	4.51	8.71E-05	***			
statusp	0.3347	0.08175	4.094	0.000281	***	statusp	0.3347	0.0818
surface1fn	0.225	0.08175	2.752	0.009802	**	surface1fn	0.225	0.0818
addmedium	-0.22828	0.10013	-2.28	0.029648	*	addmedium	-0.2283	0.1001

addplasma	-0.11742	0.10013	-1.173	0.249846		addplasma	-0.1174	0.1001
**integrin alpha5**							marginal effects	
	Estimate	Std. Error	t value	Pr(>|t|)		factor	AME	SE
(Intercept)	0.42684	0.07792	5.478	5.46E-06	***			
statusp	0.10493	0.0697	1.506	0.14232		statusp	0.1049	0.0697
surface1fn	0.20743	0.0697	2.976	0.00562	**	surface1fn	0.2074	0.0697
addmedium	0.22961	0.08536	2.69	0.01141	*	addmedium	0.2296	0.0854
addplasma	0.27851	0.08536	3.263	2.69E-03	**	addplasma	0.2785	0.0854
**integrin alphaE**							marginal effects	
	Estimate	Std. Error	t value	Pr(>|t|)		factor	AME	SE
(Intercept)	0.39608	0.09323	4.248	0.000182	***			
statusp	-0.24796	0.08339	-2.973	0.005656	**	statusp	-0.248	0.0834
surface1fn	0.37305	0.08339	4.474	9.65E-05	***	surface1fn	0.3731	0.0834
addmedium	-0.15266	0.10213	-1.495	0.145094		addmedium	-0.1527	0.1021
addplasma	-0.12886	0.10213	-1.262	0.216472		addplasma	-0.1289	0.1021
**integrin beta1**							marginal effects	

	Estimate	Std. Error	t value	Pr(>|t|)		factor	AME	SE
(Intercept)	0.05244	0.08837	0.593	0.557204				
statusp	0.21378	0.07904	2.705	0.011009	*	statusp	0.2138	0.079
surface1fn	0.31399	0.07904	3.972	0.000394	***	surface1fn	0.314	0.079
addmedium	0.02803	0.09681	0.29	0.774108		addmedium	0.028	0.0968
addplasma	0.29414	0.09681	3.038	0.004797	**	addplasma	0.2941	0.0968
**integrin beta7**							marginal effects	
	Estimate	Std. Error	t value	Pr(>|t|)		factor	AME	SE
(Intercept)	0.33571	0.13051	2.572	0.0151	*			
statusp	-0.10492	0.11674	-0.899	0.3757		statusp	-0.1049	0.1167
surface1fn	0.24192	0.11674	2.072	0.0466	*	surface1fn	0.2419	0.1167
addmedium	0.08275	0.14297	0.579	0.5669		addmedium	0.0828	0.143
addplasma	-0.05177	0.14297	-0.362	0.7197		addplasma	-0.0518	0.143

Supplementary Video Legends

Supplementary Video S1. Time-lapse video. Representative tracks of sorted CTL migration in 2D (patient 1) – fibronectin coating. The inter-frame interval is 30 seconds.

Supplementary Video S2. Time-lapse video. Representative tracks of sorted CTL migration in 2D (patient 1) – plastic with no coating. The inter-frame interval is 30 seconds.

Supplementary Video S3. Time-lapse video. Representative tracks of sorted CTL migration in 2D (patient 1) – fibronectin + conditioned medium from atherosclerotic plaque coating. The inter-frame interval is 30 seconds.
